# Inversion of the Uterus after Six Months, and Fifteen Years

**Published:** 1858-09

**Authors:** James P. White


*Inversion of the Uterus successfully Reduced after Six Months.*
By Prof. James P. White.

We have the gratification of reproducing in this number, an article with the above title, from the American Journal of the Medical Sciences, of which it is not too much to say that it will mark an important epoch in the history of obstetrical surgery. We cannot introduce the few remarks which we propose to make, better than by quoting a passage from a letter written by one of the most eminent of the obstetricians of this or any country. The name of our correspondent is withheld, inasmuch as the letter was not written for publication. He says: "I see by the last number of the American Journal, that our friend, Dr. White, has performed almost a miracle of obstetric surgery, by replacing an inverted uterus of six months standing." Our readers need not be told that, hitherto, when inversion has taken place, and the process of involution, or restoration of the normal size, has ensued, reduction has been deemed impossible. The process of involution is completed after a few weeks; consequently, nothing remains for the unfortunate patient who has suffered this accident, but the hazardous alternative of extirpation on the one hand, and, on the other hand, continuance of the distressing evils incident to inversion, during the remainder of her life. And of physical ills, few are more truly distressing than this. A constant source of annoyance and irritation, giving rise to frequent hæmorrhages and unceasing leucorrhœa, the affection renders the unhappy woman loathsome alike to herself and to others. Exhaustion and anæmia soon render her incompetent for the active duties of life, and its enjoyments. This condition has been regarded as hopeless; and yet, there may be, at least at the outset, no actual disease, the only obstacle to the recovery of comfort and strength, and even of the uterine function, being the supposed impossibility of reduction. Thanks to the case reported by Prof. White, reduction, after the lapse of an indefinite period, is no longer to be considered as impossible, but in a large proportion of instances practicable. If this be true, the occurrence of inversion henceforth does not necessarily expose the patient to the terrible calamity of a permanently irreducible uterus, even if, from any cause, the reduction be not effected prior to the completion of the process of involution. We repeat, then, that the case reported by Prof. White will mark an important epoch in the history of obstetrical surgery.

The number of the American Journal which contains Prof. White's article, also contains an account of complete inversion of the uterus of nearly twelve years' duration, successfully treated by Dr. Tyler Smith, of London. The account of this case, published originally in the London Med. Times and Gazette, may be found in the eclectic department of the present number of this Journal. The report of the case by Dr. Smith, nearly simultaneously with Prof. White's report, is a striking coincidence. Dr. Smith's case was reported to the Royal Medical and Chirurgical Society, April 13th, 1858. Prof. White communicated an account of his case at a meeting of the Buffalo Medical Association, April 6, 1858. The latter has, therefore, here, the priority by a week. The report by Prof. White was embraced in the abstract of the proceedings of the Buffalo Association, published in the number of this Journal for June, 1858; that by Dr. Smith was published in the Medical Times and Gazette, April 24th, 1858. So far as concerns the inception and completion of the undertaking, each acted, of course, independently of the other, and to both belongs the merit of originality. We propose, however, to direct attention to several points relating to the theory and practice of the operation in the two cases, in view of which we think that we may fairly claim in behalf of the article of our countryman and colleague, an importance, beyond the mere fact of success, which does not belong to the report of Dr. Smith.

The difference as regards the time that had elapsed after inversion in the two cases, is a point which will be likely to strike first the attention of the reader. This point, however, in reality, is of small importance. After involution is completed, and the uterus has remained for some months in an inverted condition, it matters little, so far as the success of the operation is concerned, whether the reduction be undertaken at the end of half a year or twenty years.* The uterus undergoes no change with the lapse of time, provided there does not occur superinduced disease resulting in adhesions which render the inversion irreducible. Prof. White, in his article, distinctly avows the belief that well-directed efforts, when there are no adhesions, will succeed, no matter how much time may have elapsed since inversion occurred.

The mechanical principles relied upon in the two cases, are quite different. Dr. Smith resorted to preparatory steps, consisting of the introduction of the hand into the vagina night and morning, squeezing and moulding the uterus

---

* *Vide* postscript to this article, p. 254.

for about ten minutes at a time. In the intervals between these manipulations, the vagina was distended and firm pressure exerted upwards by a large air pessary. The reinversion was accomplished on the eighth day from the commencement. Dr. Smith asserts that "no amount of force will suddenly reduce a case of chronic inversion." He reiterates this assertion, repeatedly saying that "the os uteri was so small that it would have been impossible to have done it at one, or even at several sittings." He adds that "He believed he could not have returned the uterus by pressure with the hand alone." He accounts for the successful result by what he terms "a process of development, rather than by the operative pressure." He believes that by the air or fluid pressure, the fundus and body of the uterus were converted into a wedge, and thus the os uteri was slowly enlarged so as to admit of reinversion. Prof. White, on the contrary, proves by the successful result in his case, that the reduction of chronic inversion may be suddenly effected: in other words, effected by properly directed efforts, in a short time; that pressure with the hand alone will effect it even at a single sitting. His observation shows conclusively that the reduction does not take place by the body and fundus acting as a wedge. Operating with the hand, he was able to determine with respect to this point, and he states that no depression was made upon the fundus; it did not dimple, and was not reflected upon itself during the operation. He accounts for the success by the effect of well-directed pressure in everting or pulling open, first the mouth, then the neck, and finally, causing the body to double upon itself. We would commend to the careful attention of the reader the explanation given in Prof. White's article, and illustrated by the diagrams introduced. As it can be at once referred to by turning to the article, it is superfluous to dwell upon it here. That it is not only philosophical, but the correct explanation, we think the reader can hardly doubt. It is important as furnishing a key both to the understanding and the proper performance of successful manipulations for the reduction of chronic inversion of the uterus.

The use of chloroform is a point in which the two cases differ. It was resorted to in Prof. White's case, but he is by no means certain that it renders the reduction more easily accomplished. Dr. Smith did not employ chloroform, owing to the feeble state of the circulation in his case. In the discussion, however, which followed the report of his case, he expresses himself unfavorable to its use, inasmuch as he had no confidence in reduction being effected except by mechanical pressure continued for several days. The importance of this agent in the present application, is to be determined by farther experience. During the discussion of Dr. Smith's case, Dr. Mc-

Kenzie stated that cases had been reported in various medical journals, in which reposition had been effected, under chloroform, without difficulty, by mere manipulation, after periods averaging from three months to two years. We think that Dr. McKenzie, in making this oral statement, was bound to make it good by citing the journals in which these cases are reported. Till this is done, we must regard the statement as indicating a loose manner of speaking, which is too common in medical discussions.

In conclusion, we congratulate the profession of this country, and especially our brethren of this city, on the article by Prof. White, which will secure for its author well-deserved and lasting distinction, and, what is of much more consequence, enlarge the humane applications of the art of medicine. *

P. S.—Since the foregoing article was in type, Dr. White has reduced an inverted uterus of fifteen years' standing! We had the pleasure of being present at the operation. The reduction was effected, under chloroform, in forty minutes. This case, a report of which will hereafter appear, confirms the correctness of the statement made in the foregoing article, that the long duration of the inversion is no obstacle to the reduction. The case also shows, conclusively, the correctness of Dr. White's views as to the rationale of reduction, in opposition to the views of Dr. Tyler Smith. We renew our congratulations on the successful result in this case. *

